# Non-coding RNAs in Regulating Tumor Angiogenesis

**DOI:** 10.3389/fcell.2021.751578

**Published:** 2021-09-20

**Authors:** Xin Song, Yanan Guo, Peng Song, Dongzhu Duan, Wenjing Guo

**Affiliations:** ^1^School of Life Sciences and Engineering, Lanzhou University of Technology, Lanzhou, China; ^2^School of Traditional Chinese and Western Medicine, Gansu University of Traditional Chinese Medicine, Lanzhou, China; ^3^Affiliated Hospital of Gansu University of Chinese Medicine, Lanzhou, China; ^4^Key Laboratory of Prevention and Treatment for Chronic Diseases by TCM, Lanzhou, China; ^5^Shaanxi Key Laboratory of Phytochemistry and College of Chemistry and Chemical Engineering, Baoji University of Arts and Sciences, Baoji, China

**Keywords:** ncRNA, tumor metabolism, tumor angiogenesis, molecular mechanism, biological function

## Abstract

Non-coding RNAs (ncRNAs) are RNAs that do not encode proteins, but perform biological functions in various physiological and pathological processes, including cancer formation, inflammation, and neurological diseases. Tumor blood vessels are a key target for cancer management. A number of factors regulate the angiogenesis of malignant tumors. NcRNAs participate in the regulation of tumor angiogenesis. Abnormal expression of ncRNAs act as tumor suppressors or oncogenes to affect the development of tumors. In this review we summarized the biological functions of ncRNAs, and discussed its regulatory mechanisms in tumor angiogenesis. This article will provide new insights for the research of ncRNAs in tumor angiogenesis.

## Highlights

- Biological functions of ncRNAs were comprehensively discussed.
- The association of ncRNA and tumor angiogenesis was illustrated.
- The mechanisms of ncRNAs in regulating angiogenesis in tumor development were summarized.

## Introduction

It is well-acknowledged that cancer is one of the most severe diseases which leads to death and cripples life expectancy worldwide. Cancer therapy targeting solely tumor cells has been identified as the most extensive and efficient approaches in the past, but the clinical limitations still exist, such as toxic side effects (Montgomery et al., 2019). Since the rapid proliferation of tumor cells need new vascular systems to supply nourishment, angiogenesis plays a critical role in tumor maintenance, metabolic disorder, and tumor tissues dissemination/metastasis (Folkman, 2002). Therefore, anti-tumor angiogenesis treatment has emerged as an appealing solution in recent years.

Angiogenesis has been defined as a progression that new blood vessels are regenerated from the existing capillary network. An “angiogenic switch” is always activated in tumors, thus causing continuous new vessels generation. Tumor-associated neovasculature is a complex physiological incident, which is governed by a variety of pro- or anti-angiogenic cytokines and multiple signaling pathways, such as vascular endothelial growth factor (VEGF; Apte et al., 2019), angiopoietin (Carbone et al., 2018), etc.

As a class of significant RNA, non-coding RNAs (ncRNAs) are capable of performing biological functions at the RNA level. In high-grade organisms, up to half of DNA is transcribed into RNA, most of which are ncRNAs. It can be indicated that ncRNAs exert a key role in organismal development. Numerous studies have shown that ncRNAs participate in the occurrence and processing of tumors by functioning as oncogenes or tumor suppressor genes (Anastasiadou et al., 2018) thus these RNAs can be used as diagnostic and prognostic markers for cancer patients. New information has proved that a large number of ncRNAs involve in the modulation of tumor angiogenesis. NcRNAs [microRNA (miRNA), long ncRNA (lncRNA), circular RNA (circRNA), small interfering RNA (siRNA), etc.] can interact with various angiogenic factors (VEGF, MMP2, etc.) and regulate signal pathways, such as Akt pathway and ERK1/2 pathway, in tumors (Safa et al., 2020; Wu et al., 2020). This article reviews and summarizes the major types of ncRNAs and their mechanisms in regulating tumor angiogenesis.

## The Biological Source and Functions of Non-Coding RNAs

**FIGURE 1:**
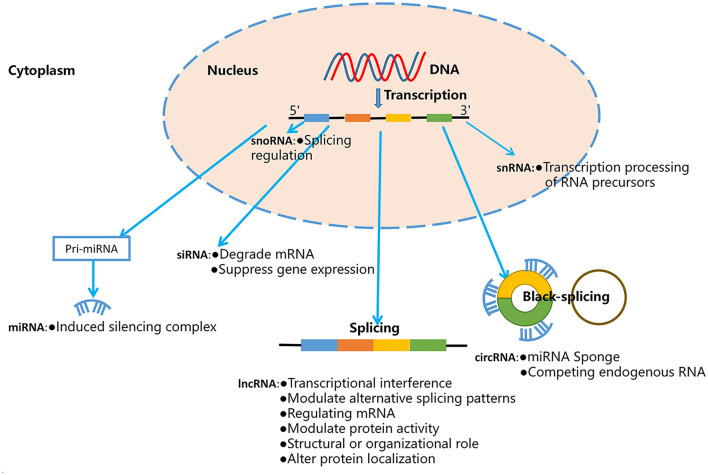
The biological source and function of ncRNAs.

Non-coding RNAs are a large and diverse class of RNAs that lack the function of encoding proteins, but perform important biological and pathological functions in many diseases, including cancers, inflammation, and others (Lekka and Hall, 2018). According to the relative molecular weight, morphology and function, ncRNAs are classified into miRNAs, circRNAs, and lncRNAs, Piwi-interacting RNAs (piRNAs), small nuclear RNAs (snRNAs), and small nucleolar RNAs (snoRNAs; Cheng et al., 2020). The biological source and function of these ncRNAs as shown in Figure 1. Previous studies show that ncRNAs can mediate a variety of fundamental cellular processes, such as differentiation, proliferation, apoptosis, angiogenesis, and cell metabolism through regulating gene expression and signaling pathways (Wei et al., 2020). Therefore, ncRNAs can act as oncogenes or tumor suppressor genes, and biomarkers and therapeutic targets of multiple malignancies.

### MicroRNAs

MicroRNAs are a type of eukaryotic endogenous small RNAs with a length of 18–25 nucleotides. Its main function is to regulate gene expression by binding to targeted RNAs (Beermann et al., 2016). The formation of miRNAs can be divided into two processes. First, the miRNA gene is transcribed into the initial transcription product (pri-miRNA), the pri-miRNA is recognized by the microprocessor and cut by the RNase III Drosha to form a hairpin structure called precursor miRNA (pre-miRNA), which is transferred from the nucleus to the cytoplasm under the mediation of Exportin-5 and RAN-GTP. Second, the pre-miRNA is cleaved into double-stranded miRNA under the action of RNase III Dicer. It then interacts with Argonaute to assemble the miRNA-induced silencing complex. The mature miRNA remains in the complex while the other strand is degraded (Ha and Kim, 2014). The mature miRNAs binds to targeted mRNA to perform post-transcriptional gene silencing role, thereby reducing the stability or inhibiting the translation of the target gene (Yang X. et al., 2020).

### Long Non-coding RNAs

Long ncRNAs, with a length of more than 200 nucleotides, is transcribed by RNA polymerase II and involved in a variety of biological processes (Wong et al., 2018). LncRNAs exists both in the nucleus and cytoplasm, perform different functions according to its subcellular location (Kopp and Mendell, 2018; Miao et al., 2019; Li S. et al., 2020). The upstream promoter region encoding the protein interfered with the expression of downstream genes, and inhibits RNA polymerase II or recruits mediator proteins and chromatin remodeling enzymes to affect downstream gene transcription (Cho et al., 2018). LncRNA can be used as a scaffold to recruit RNA-binding proteins to form a nucleic acid-protein complex, and participate in chromatin remodeling and transcriptional regulation (Ban et al., 2020). In addition, it can also form a complementary double-strand with mRNA to interfere with the shearing of mRNAs (Romero-Barrios et al., 2018). Furthermore, lncRNAs interact with the protein bound to the 3′untranslated region (UTR) of mRNA in the cytoplasm to regulate the stability of mRNA (Sun et al., 2020). Antisense lncRNAs regulate the stability of mRNA by forming a double strand with mRNA (Liu et al., 2014). When lncRNAs is used as competing endogenous RNAs (ceRNAs), it can bind to miRNAs and prevent itself from inhibiting its targeted mRNAs (Li H. et al., 2020). Moreover, it can positively or negatively regulate protein translation, encoding micropeptides with regulatory functions, and can also regulate signaling pathways in the cytoplasm, and bind to specific proteins to change the cellular localization of proteins (Hosen et al., 2018).

### Circular RNAs

Circular RNAs have a closed-loop structure and is more stable than linear RNAs (Hanahan and Weinberg, 2011; Lee et al., 2020). In recent years, many circRNAs with important functions are coming to light and researchers began to focused on the properties and functions of circRNAs (Iurca et al., 2020). CircRNAs is divided into three types: exonic circular RNA (ecRNA), circular intronic RNA (ciRNA), and exon-intron circular (EIciRNA; Guo et al., 2014). Among them, ecRNA is the most common one, which is mainly locate in the cytoplasm, while ciRNA and EIciRNA are abundant in the nucleus (Liang and Wilusz, 2014). CircRNAs have been demonstrated as endogenous competitive RNAs that bind to miRNAs to inhibit targeted mRNA expression (Su et al., 2019).

### Small Interfering RNAs

Small interfering RNAs are a kind of small double stranded RNA (dsRNA) with a length of 20–25 nucleotides, that are made from fully complementary long double-stranded RNA through dicer shearing (Leung et al., 2016). Exogenous dsRNA is cleaved by Dicer enzyme and TAR-RNA binding protein to form siRNA, then the siRNA loaded onto the Argonaute protein (AGO2) to form an RNA-induced silencing complex (RISC). RISC and targeted mRNA are partially or completely complementary paired, turning double-stranded siRNA into single-stranded siRNA. After combining with single-stranded siRNA, RISC become an active RISC. Targeting mRNA can be degraded through combining with activated RISC (Singh et al., 2018).

### Other Non-coding RNAs

Piwi-interacting RNAs are small single-stranded RNAs with a length of 24–32nt. They have strong sense and antisense strand specificity, and the first nucleotide at the 5′-end is uracil-prone, while the 3′-end is modified by 2′-O-methylation. This type of end modification can prevent the degradation of mature piRNA genes. PiRNA must interact with PIWI protein form a piRNA silencing complex to play its regulatory role. Current studies (Guo et al., 2020) have shown that piRNA and PIWI abnormally expressed in gastric cancer, breast cancer, kidney cancer, colon cancer and lung cancer, and are involved in the occurrence, development and metastasis of cancers. PiRNAs could be potential prognostic and diagnostic biomarkers, and cancer treatment targets. PiRNA clusters are mainly distributed around centromeres and subtelomeres. They are transcribed into precursor piRNAs by RNA PolII, and are transported to the cytoplasm through primary processing pathways to form primary piRNAs, the pre-processed antisense piRNAs are loaded on Aub (Aub belongs to the Piwi subfamily) in the cytoplasm, and target the sense reverse transcript, resulting in the production of sense piRNA. These sense piRNAs are loaded onto Argonaute 3 (AGO3), and process the precursor antisense piRNA into mature piRNA. This amplification cycle continues with the continuous expression of Aub and AGO3, thereby causing a large amount of piRNA in the cell to be amplified. This phenomenon is called the “Ping-Pong” cycle (Soleimani et al., 2020). PiRNA mainly binds with PIWI or AGO3 protein, a member of the PIWI subfamily, to maintain genome stability by silencing transposable elements and regulating coding mRNA (Ng et al., 2016).

Small nuclear RNAs are a class of 50–200 nucleotides small RNAs exist in the nucleus. They are the main components of eukaryotic RNA spliceosomes. SnRNA is rich in uracil and usually numbered U1–U7. All snRNAs (except U6) have a 2,2,7-trimethylated 5′-guanosine cap (Bohnsack and Sloan, 2018). In addition to regulating the correct expression of histone mRNA and the production of rRNA, it is involved in the formation of snRNAs complexes with proteins to catalyze the splicing of precursor mRNA (Guiro and Murphy, 2017).

Small nucleolar RNAs, with a length of 60–300 nucleotides located in the nucleolus, are divided into box C/D snoRNAs and box H/ACA snoRNAs. Box C/D snoRNAs mainly mediate 2′-O-methylation at specific sites of rRNA, box H/ACA snoRNA mainly mediate the pseudo uridylation of rRNA specific sites. Both can combine with ribonucleoprotein to play a key role in rRNA processing, participate in the splicing process of rRNA and other small RNA genes (Gong et al., 2017).

## Tumor Angiogenesis

Tumors need to rapidly develop new vascular networks to support the rapid proliferation of cancer cells. Angiogenesis, which is defined as regeneration of new blood vessels from the existing capillary network, participates in the entire process of tumor development (Liu et al., 2011). Relevant studies have shown that solid tumors cannot grow to more than 2–3 mm without inducing their own blood supply. This view explains the association between angiogenesis and tumor development (Liu et al., 2016). Therefore, angiogenesis plays an important role in the occurrence and development of tumors. In order to develop new drugs for anti-tumor angiogenesis, it is necessary to better understand the cellular and molecular mechanisms involved in tumor angiogenesis. Tumor angiogenesis is a complex process, including degradation of basement membrane, proliferation and migration of endothelial cell, and other steps.

As the key pro-angiogenic factors, VEGF and its receptors play a vital role in the whole process of tumor angiogenesis. VEGF family members are able to mediate a series of intracellular signal transduction pathways activation by fully binding to three significant tyrosine kinase receptors (VEGFR1, VEGFR2, and VEGFR3) and act on endothelial cells, which results in cell mitosis and capillary formation (Holmes et al., 2007). After combining with VEGFR2, several VEGF family members can significantly stimulate the differentiation and proliferation of vascular endothelial cells, promote angiogenesis and enhance the permeability of capillaries (Chang et al., 2009). VEGFR2 regulates the expression of related genes through PLC-γ-MEK- MAPK pathway, which leads to EC proliferation (Downward, 2004). VEGFR-2 can modulate cell migration by activating PI3K pathway. The activation of PI3K/Akt pathway in tumor microenvironment can inhibit endothelial cell apoptosis, ensure ECs survival and contribute to angiogenesis. It has been verified that PI3K/Akt pathway regulate hypoxia inducible factor-1 (HIF-1) and VEGF expression by activating kinases p70S6K1 and HDM2 in tumor tissue (Skinner et al., 2004; Jiang and Liu, 2008). Moreover, PTEN can inhibit tumor angiogenesis by promoting PI3K/Akt/VEGF/eNOS signaling pathway (Ma et al., 2009).

**FIGURE 2:**
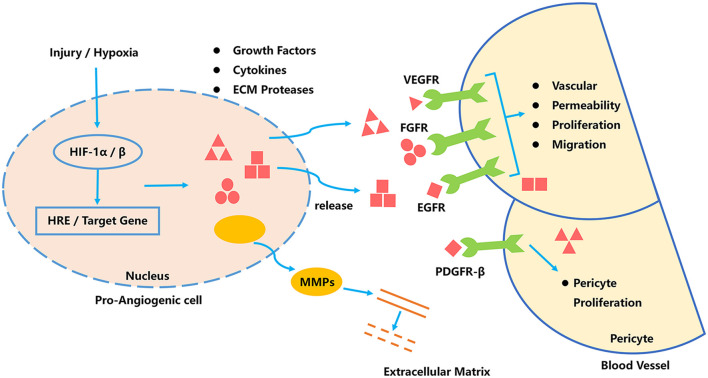
Signaling pathways of tumor angiogenesis.

Tumor angiogenesis is a complex process, depends on the synergistic effect of multiple regulatory factors (Sahraei et al., 2019). Tumor internal environment can induce HIF-1 activating cells, to release a large amount of vascular endothelial growth factor-A (VEGF-A), vascular endothelial growth factor-2 (VEGF-2), fibroblast growth factor-2, and stromal cell-derived factor-1α/β (SDF-1α/β). These factors stimulate blood vessel formation and remodeling (Hu et al., 2019). In addition, pre-stimulation-angiogenic cells secrete matrix metalloproteinases (MMPs) to digest the basement membrane and accelerate vascular remodeling. The stability of the vascular network is an important factor that influence the development of tumors. The platelet particles release PDGF-BB and transforming growth factor-α (TGF-α), angiopoietin-1 (ANG-1) to promote the stability and maturity of the complex vascular network. Signaling pathways of tumor angiogenesis as shown in Figure 2.

## Non-Coding RNAs to Regulate Tumor Angiogenesis

### MicroRNAs Regulate Tumor Angiogenesis

MicroRNAs play an important role in various biological processes, and their roles in the pathogenesis of diseases have been observed (Jia et al., 2016). Studies have shown that miRNAs, such as miR-21, miR-106a, miR-126, miR-155, miR-182, miR-210, and miR-424, are important factors that regulate tumor angiogenesis (Huang and Chu, 2014; Jing et al., 2017; Alhasan, 2019; Sun et al., 2019; Wu et al., 2019). MiR-21, miR-126 (Du et al., 2014; Alhasan, 2019), and miR-93 (Fang et al., 2011, 2012; Fabbri et al., 2015; Liang et al., 2017; Ashrafizadeh et al., 2020) enhance the expression of HIF-1 and VEGF through targeting PTEN and inhibiting the expression of angiogenesis inhibitor thrombospondin-1 (THBS1). MiRNAs can combine with lncRNAs, such as MALAT1, to inhibit large tumor suppressor 2 (LATS2) to regulate the growth, invasion and metastasis of tumor cells. The common microRNAs that regulate tumor angiogenesis are shown in Table 1.

**TABLE 1:** Common microRNA targets and functions in regulating tumor angiogenesis.

**MicroRNA**	**Expression in tumor**	**Target**	**Function**	**References**
miR-21	Upregulation	PTEN	Targeting PTEN induces tumor angiogenesis, activates AKT and ERK1/2 signaling pathways, thereby enhancing the expression of HIF-1 and VEGF.	Liu et al., 2011
		STAT3	Knockout of STAT3 gene can reduce the level of miR-21 excised body and reduce the level of VEGF, thereby blocking angiogenesis.	Liu et al., 2016
		TAMs	Loss of miR-21 expression results in macrophages (TAMs) biased toward the pro-inflammatory vascular inhibitory phenotype, reducing tumor formation and inducing tumor cell death.	Sahraei et al., 2019
		THBS1	Inhibits the expression of angiogenesis inhibitor thrombospondin-1 (THBS1) in the receptor EC.	Hu et al., 2019
		TGFBI, COL4A1	Directly targeting and inhibiting TGFBI and COL4A1, increases the formation of endothelial cells.	Wu et al., 2019
miR-126	Downregulation	VEGF	Experimental studies of liver cancer cells inoculated in nude mice showed that the level of VEGF and the positive rate of VEGF were lower in the miR-126 overexpression group, but higher in the miR-126 inhibition group, miR-126 inhibited liver cancer angiogenesis.	Jing et al., 2017
		VEGFA	miR-126 inhibits the proliferation of MCF7 cells, induces apoptosis, and inhibits tumor angiogenesis by downregulating the VEGF-A signaling pathway.	Alhasan, 2019
		VEGF	Carcinoma interstitial crosstalk induces miR-126 inhibition and promotes angiogenesis and invasive growth of cervical cancer.	Huang and Chu, 2014
		VEGFA	Sponge miR-126-5p promotes the expression of VEGFA, nasal mucus protein and TWIST, thereby promoting the metastasis of colorectal cancer.	Sun et al., 2019
		LRP6, PIK3R2	Overexpression of miR-126-3p *in vivo* inhibits the formation of endothelial cell capillaries, significantly reducing tumor volume and microvessel density.	Du et al., 2014
miR-93	Upregulation	VEGF	It can increase the angiogenesis ability of human umbilical vein endothelial cells (HUVECs), thereby improving blood vessel density, increasing proliferation and migration, and promoting lumen formation and sprouting.	Ashrafizadeh et al., 2020
		EPLIN	MIR-93 promotes tumor angiogenesis by reducing the expression of EPLIN.	Liang et al., 2017
		LATS2	miR-93 promotes tumor angiogenesis and metastasis by inhibiting the expression of LATS2.	Fang et al., 2012
		VEGF, IL-8	Reversely regulate VEGF and IL-8 gene expression and protein release, MCP-1 and PDGF also have potential regulatory effects.	Fabbri et al., 2015

#### MiR-21

MiR-21 is encoded by a gene containing miR-21 in the intron region of the TMEM49 gene (Ma et al., 2011). The primary transcript, pri-miR-21, is produced by RNA polymerase II transcription in the nucleus and processed into mature miR-21 in the cytoplasm (Liu et al., 2018). MiR-21 is involved in almost every aspect of tumor growth, such as promoting cell proliferation, invasion and metastasis, genome instability and mutation, inflammation, replication immortalization, metabolic abnormality, angiogenesis, evasion of apoptosis, immune destruction, and growth inhibition (Pfeffer et al., 2015). Studies have shown that miR-21 induces tumor angiogenesis by targeting PTEN and activates the AKT and ERK1/2 signaling pathways, thereby enhancing the expression of HIF-1 and VEGF (An et al., 2019). HIF-1 is a key target of miR-21 in regulating tumor angiogenesis. Liu et al. (2016) have shown that miR-21 leads to signal transducer and activator of transcription-3 (STAT3) activation and increases the level of VEGF in recipient cells, thereby promoting angiogenesis. Sahraei et al. (2019) suggested that the overexpression of miR-21 in tumor-associated macrophages (TAMs) led to the overall reorganization of the transcriptional regulatory network, which favored the formation of pro-inflammatory blood vessels, and promote tumor angiogenesis and tumor cell growth. In addition, miR-21-5p is highly enriched in endothelial progenitor cells-exosomes, and specifically inhibits the expression of angiogenesis inhibitor thrombospondin-1 (THBS1; Hu et al., 2019). Wu et al. (2019) found that under hypoxic conditions, the expression of miR-21-5p in the exosomes of papillary thyroid carcinoma BCPAP cells was significantly up-regulated. MiR-21-5p directly target and inhibit TGFBI and COL4A1, increasing endothelial cell proliferation, promoting tumor angiogenesis.

#### MiR-126

MiR-126 is encoded by a single gene located in the intron of the encoding protein 7 with an EGF-like domain (EGFL7) and is located on chromosome 9q34.3. MiR-126 is considered to be one of the most important miRNAs for maintaining the integrity of blood vessels, of which both miR-126-3p and miR-126-5p have biological activity (Casciaro et al., 2018). Jing et al. (2017) found that miR-126 inhibited tumor angiogenesis by downregulating the VEGF-A signaling pathway. In addition, miR-126 acts as a tumor suppressor in breast cancer cells, blocking tumor cell growth and metastasis by inhibiting tumor angiogenesis (Alhasan, 2019). Inhibition of miR-126 can induce the upregulation of the pro-angiogenic gene adrenomedullin to promote angiogenesis of cervical cancer (Huang and Chu, 2014). In colorectal cancer (Sun et al., 2019), the oncogene YAP1 forms a complex with β-catenin/TCF4, which binds to the MALAT1 promoter and miR-126-5p, promotes the expression of VEGFA, SLUG and TWIST, and regulates the angiogenesis of colorectal cancer. Du et al. (2014) found that that miR-126-3p significantly inhibits HCC cell migration and invasion of extracellular matrix, and inhibits capillary formation of endothelial cells *in vitro*. Overexpression of miR-126-3p significantly reduced tumor volume and microvessel density *in vivo*, and LRP6 and PIK3R2 were the targets. The level of miR-126-3p is negatively correlated with LRP6 and PIK3R2 in HCC tissues. In addition, rescue experiments showed that the function of angiogenesis of miR-126-3p is mediated by LRP6 and PIK3R2.

#### MiR-93

MiR-93 is involved in angiogenesis and tumor growth. Experimental studies have shown that miR-93 promotes tumor angiogenesis by reducing the expression of EPLIN (Ashrafizadeh et al., 2020). Upregulation of miR-93-5p can increase the angiogenic ability of HUVECs, thereby improving blood vessel density, increasing proliferation and migration of cancer cells (Liang et al., 2017). Fang et al. (2011) showed that overexpression of miR-93 can promote the proliferation, growth, migration and tube formation of endothelial cells, induce blood vessel formation, and extend blood vessels to tumor tissues at high density. The expression of miR-93 enhances the survival and invasive ability of cells, promotes tumor angiogenesis and metastasis by inhibiting the expression of LATS2. The formed tumor is rich in blood vessels (Fang et al., 2012). Fabbri et al. (2015) found that in the glioma cell lines U251 and T98G, pro- and antago-miR-93 can reversely regulate the expression of VEGF and IL-8 genes and protein release, which is associated with angiogenesis in glioma.

### Long Non-coding RNAs Regulate Tumor Angiogenesis

Abnormal expression of lncRNAs has been observed in many human cancers, and their role as tumor suppressors or oncogenes is associated with the staging and grading of tumors (Bhan et al., 2017). Different lncRNAs play different roles in tumor regulation. LncRNAs such as HOTAIR (Li et al., 2014; Yu and Li, 2015; Fu et al., 2016; Zhou et al., 2020), MALAT1, ANRIL and SRA are up-regulated in tumors and play the role of oncogenes, while MEG3, GASS and LncRNA-p21 are down-regulated in tumors and play a role of tumor suppressors. H19 (Conigliaro et al., 2015; Jia et al., 2016; Jiang et al., 2016; Lv et al., 2017; Yuan et al., 2019; Liu Z.Z. et al., 2020) plays both oncogene and suppressor roles in tumors. LncRNA H19, HOTAIR, and MVIH (Yuan et al., 2012; Lei et al., 2016; Wang Y. et al., 2020) mainly regulate the proliferation, migration, invasion and angiogenesis of tumor cells by regulating VEGF, VASH2, and miR138/HIF1α axis. LncRNAs that regulate tumor angiogenesis are shown in Table 2.

**TABLE 2:** Common lncRNA targets and functions in regulating tumor angiogenesis.

**LncRNA**	**Expression in tumor**	**Targets**	**Functions**	**References**
H19	Upregulation	VEGF	Up-regulate the production and release of VEGF and enhance the ability of HUVEC cells to arrange tubular structures *in vitro*.	Conigliaro et al., 2015
		VASH2	Proliferation, migration and tubular formation of vascular endothelial cells. After knockout, the expression of miR-29a is up-regulated to reduce the expression of VASH2.	Jia et al., 2016
		DNMT3B	Promote the proliferation, invasion and migration of bladder cancer cells, regulate epithelial-mesenchymal transition (EMT) and rearrange the cytoskeleton.	Lv et al., 2017
		VASH1	Knockout inhibits the ability of the promoter region of VASH1 to recruit methyl groups, increases the expression of VASH1 and the secretion of HAMSCs, thereby inhibiting angiogenesis.	Yuan et al., 2019
		HIF-1α	Promote tumor cell proliferation, migration, invasion and angiogenesis through the miR138/HIF1α axis.	Liu Z.Z. et al., 2020
HOTAIR	Upregulation	VEGF	Inhibit cell apoptosis, stimulate angiogenesis, accelerate cell cycle progress, and induce epithelial-mesenchymal transition (EMT).	Zhou et al., 2020
		VEGFA	Promote angiogenesis through GRP78-mediated upregulation of VEGFA and Ang2 expression.	Fu et al., 2016
		VEGFC	Promote angiogenesis of breast epithelial cells through transcriptional activation of VEGF-C, thereby promoting the occurrence of metastasis.	Li et al., 2014
MVIH	Upregulation	VEGF	Activate angiogenesis in mouse models to promote tumor growth and intrahepatic metastasis.	Yuan et al., 2012
		Ki67	The expression level of MVIH in breast cancer tissue is higher than that in adjacent tissues, and the high expression of MVIH is closely related to the expression of Ki67.	Lei et al., 2016
		PGK1	The interaction between RPS24c mRNA and LncRNA MVIH activates colorectal cancer angiogenesis by inhibiting the secretion of PGK1.	Wang Y. et al., 2020

#### H19

The H19 gene is mainly expressed in endoderm and mesoderm-derived tissues and locates on human chromosome 11p15.5. Its expression is down-regulated after birth (Yang et al., 2021). Studies have found that upregulation of H19 is related to angiogenesis (Rolla et al., 2021). Conigliaro et al. (2015) found that H19, which is highly expressed in CD90 + Huh cells, enters endothelial cells through exosomes to up-regulate the production and release of VEGF, which ultimately promotes angiogenesis and affects its tumor microenvironment. Jia et al. (2016) found that the upregulation of H19 in glioma tissues and glioma-associated endothelial cell (GEC) microvessels can promote the proliferation, migration and tubular formation of vascular endothelial cells in gliomas. H19 gene targets the 3′-UTR region of angiostatin 2 (VASH2) by inhibiting the expression of miR-29a. H19 is significantly overexpressed in glioblastoma tissues and promotes the angiogenesis of cells *in vitro* (Jiang et al., 2016; Lv et al., 2017). Yuan et al. (2019) found that H19 can interact with histone methyltransferase enhancer 2 (EZH2) to promote angiogenesis. Liu Z.Z. et al. (2020) found that the up-regulated H19 in glioma cells can promote tumor cell proliferation, migration, invasion, and angiogenesis through the miR138/HIF1α axis.

#### HOX Transcript Antisense Intergenic RNA

HOX transcript antisense intergenic RNA (HOTAIR) contains more than 2,100 nucleotides and locates in the 12q13.13 region of chromosome. It has a *trans*-acting (Cai et al., 2014) and promoting effect on the proliferation, metastasis, angiogenesis and metabolism of cancer cells (Yu and Li, 2015). In cervical cancer patients, elevated HOTAIR levels are significantly associated with poor prognosis. Fu et al. (2016) found that HOTAIR promotes tumor cell growth and angiogenesis by directly activating VEGFA and Ang2 expression in nasopharyngeal carcinoma cells. Li et al. (2014) found that in metastatic breast cancer, HOTAIR promotes the angiogenesis of breast epithelial cells through transcriptional activation of VEGF-C, thereby promoting tumor metastasis.

#### MVIH

MVIH locates in the intron region of the ribosomal protein S24 gene (RPS24) and overlaps the exons of RPS24 (Wang Y. et al., 2020). MVIH is associated with microvascular invasion of liver cancer. Yuan et al. (2012) found that in liver cancer, MVIH promotes tumor growth and intrahepatic metastasis by activating angiogenesis. Wang Y. et al. (2020) found that MVIH inhibits the secretion of PGK1 to activate colorectal cancer angiogenesis through interacting with RPS24 (ribosomal protein S24), which is highly expressed in colorectal cancer.

### CricRNAs Regulate Tumor Angiogenesis

CircRNAs act as signaling molecules in regulating tumor growth, angiogenesis, invasion, metastasis, and chemotherapeutic sensitivity. In addition, circulating exosome circRNAs can affect tumor progression and malignant characteristics. CircRNA has great value in tumor diagnosis and prognosis, and is a promising non-invasive biomarker (Wang M. et al., 2020). Circ-ATXN1 (Liu X. et al., 2020), circ-SHKBP1 (Xie et al., 2020), and circ-001971 (Chen et al., 2020) mainly regulate VEGF through sponging miRNAs and activate PI3K/AKT signaling pathway to promote tumor cell proliferation, migration and angiogenesis. Common circRNAs that regulate tumor angiogenesis are shown in Table 3.

**TABLE 3:** Common cricRNA targets and functions in regulating tumor angiogenesis.

**CricRNA**	**Expression in tumor**	**Targets**	**Functions**	**References**
circ-ATXN1	Upregulation	MMP2, VEGFA	Knockout of circ-ATXN1 can significantly inhibit cell viability, migration and tube formation of gliomas.	Liu X. et al., 2020
circ-SHKBP1	Upregulation	VEGF	SHKBP1 acts as a sponge molecule, adsorbing miR-582-3p to increase the expression of HUR and enhance the stability of VEGF mRNA.	Xie et al., 2020
Hsa-circ-0000515	Upregulation	CXCL10	hSA-circ-0000515 can bind to miR-296-5p and prevent it from inhibiting the expression of CXCL10.	Cai et al., 2021
circ-001971	Upregulation	VEGFA	circ-001971 acts as a ceRNA to reduce the inhibition of miR-29c-3p on vascular endothelial growth factor, thereby increasing the proliferation, invasion and angiogenesis of major bowel cancer.	Chen et al., 2020
circ-0056618	Upregulation	CXCR4, VEGFA	circ-0056618 acts as a sponge molecule, adsorbs miR-206, up-regulates the expression of CXCR4 and VEGFA in colorectal cancer, and promotes cell proliferation, migration and angiogenesis.	Zheng et al., 2020
circ-PRRC2A	Upregulation	TRPM3	circ-PRRC2A acts as a sponge molecule, adsorbing miR-514a-5p and miR-6776-5p to prevent the degradation of the tissue-specific oncogene TRPM3 mRNA, and promote angiogenesis and tumor metastasis.	Li W. et al., 2020
circRNA-MYLK	Upregulation	VEGFA/VEGFR2	circ-MYLK acts as a sponge molecule, adsorbing miR-29a to release its inhibition of VEGFA, thereby activating the VEGFA/VEGFR2 signaling pathway, and promoting the proliferation, migration, tubular formation and cytoskeleton rearrangement of HUVEC.	Zhong et al., 2017
circ-DICER1	Upregulation	PI3K/AKT	circ-DICER1 acts as a sponge molecule, adsorbs miR-103a-3p/miR-382-5p, weakens its negative regulation of ZIC4 in GEC, and promotes cell viability, migration and tube formation of GEC.	He et al., 2019

Previous studies have shown that (Liu X. et al., 2020) angiogenesis plays an important role in the occurrence and development in gliomas. The expression of circ-ATXN1 significantly enhances the cell viability, migration and tube formation in GECs. Circ-ATXN1 functionally targets miR-526b-3p in the RISC and affects the angiogenesis of vascular endothelial cells by negatively regulating the expression of MMP2/VEGFA. Xie et al. (2020) found that circ-SHKBP1 is expressed in gastric cancer tissues and serum of patients. As a sponge molecule, circ-SHKBP1 can adsorb miR-582-3p to increase the expression of HUR, enhance the stability of VEGF mRNA, and promote angiogenesis of gastric cancer cells. Circ-001971 acts as a ceRNA by relieving miR-29c-3p-induced VEGFA inhibition, thereby aggravating the proliferation, invasion and angiogenesis of colorectal cancer (Chen et al., 2020). Zheng et al. (2020) found that the expression of circ-0056618 increased in colorectal cancer tissues and colorectal cancer cell lines. Circ-0056618 acts as a sponge molecule to adsorb miR-206 and eliminate the inhibitory effect of miR-206, thereby upregulating CXCR4 and VEGF-A in colorectal cancer. Circ-PRRC2A acts as a sponge molecule to adsorb miR-514a-5p and miR-6776-5p to prevent the degradation of the mRNA of tissue-specific oncogene TRPM3, promoting angiogenesis and tumor metastasis (Li W. et al., 2020). CircRNA-MYLK can directly bind to miR-29a and reduce the inhibition of VEGFA, thereby activating the VEGFA/VEGFR2 signaling pathway, ectopic expression of circRNA-MYLK promotes the proliferation, migration, tubular formation and cytoskeleton rearrangement of HUVEC (Zhong et al., 2017). Hsa-circ-0000515 is up-regulated in breast cancer tissues. Hsa-circ-0000515 binds to miR-296-5p to prevent it from inhibiting CXCL10 expression, promotes cell cycle progression, cell proliferation and invasion of breast cancer cells, and increases the potential of cancer cells to promote angiogenesis (Cai et al., 2021). He et al. (2019) showed that circ-DICER1 acts as a molecular sponge to adsorb miR-103a-3p and miR-382-5p, and weaken its negative regulatory effect on ZIC4 in GECs. ZIC4 up-regulates the expression of its downstream target Hsp90β, and Hsp90 activates the PI3K/AKT signaling pathway and promotes cell viability, migration and tubular cell formation.

### Small Interfering RNAs Regulate Tumor Angiogenesis

Small Interfering RNAs silences targeted genes to inhibit angiogenesis of cancer cells and tumor growth. *In vivo*, siRNAs can significantly influence tumor angiogenesis by regulating related genes and pathways. Common siRNAs that regulate tumor angiogenesis are shown in Table 4.

**TABLE 4:** siRNA delivery system, target and function of regulating tumor angiogenesis.

**siRNA delivery system**	**Content in tumor**	**Targets**	**Functions**	**References**
Polymeric micelles	Upregulation	HIF-1α, VEGF	Inhibit the expression of HIF-1α and VEGF in RB cells, inhibit the HIF-1α/VEGF/VEGFR signaling pathway, and the proliferation, migration, invasion of vascular endothelial cells	Yang F. et al., 2020
HA-TAT-TMC-TC NPs	Upregulation	PD-L1, STAT3	Downregulation of PD-L1 and STAT3, inhibit proliferation, migration and angiogenesis of cancer cells, inhibit tumor growth in the body.	Bastaki et al., 2021
CMNPs carrying Ang2-siRNA	Upregulation	Bax/Bcl-2, caspase-3	Inhibit tumor angiogenesis and promote cell apoptosis by adjusting the ratio of Bax/Bcl-2 and increasing the lytic expression of caspase-3	Shan et al., 2020
CL4H6-LNPs	Upregulation	STAT3, HIF-1α	Silencing STAT3 and HIF-1α leads to an increase in the concentration of macrophages (CD11b^+^ cells) and M1 macrophages that infiltrate the tumor microenvironment (CD169^+^ cells). It also leads to the reversal of the tumor-promoting function of TAMs-mainly angiogenesis and tumor cell activation.	Shobaki et al., 2020

Yang F. et al. (2020) used polymer micelles as a carrier to deliver triptolide and siRNA to retinoblastoma (RB) cells. The micelle carrier loaded with triptorelin and HIF-1 siRNA showed effective cell internalization, inhibited the expression of HIF-1α and VEGF in RB cells, leading to inhibition of the HIF-1α/VEGF/VEGFR signaling pathway, and the proliferation, migration, and invasion of vascular endothelial cells. Bastaki et al. (2021) generated trimethyl chitosan and thiolated chitosan nanoparticles (NPs) conjugated with HIV-1-derived TAT peptide and HA (hyaluronic acid). These NPs exhibited prominent physicochemical characteristics, notable siRNA encapsulation, serum stability, non-toxicity, controlled siRNA release, and extensive cellular uptake by cancer cells. The siRNAs silenced targeted genes, immune checkpoint molecule programmed cell death ligand 1 and oncogene transcription factor STAT3, which significantly inhibits the proliferation, migration and angiogenesis of cancer cells, inhibits tumor growth in the body. Shan et al. (2020) found that chitosan magnetic nanoparticles (CMNPs) carrying Ang-2 small interfering RNA plasmids have inhibitory effects on malignant melanoma, and can significantly inhibit the growth of melanoma. *In vivo*, Ang2-CMNP significantly inhibits tumor angiogenesis and promotes cell apoptosis by adjusting the ratio of Bax/Bcl-2 and increasing the expression of caspase-3. Shobaki et al. (2020) using optimized load CL4H6-LNP (CL4H6 is a novel, pH-sensitive cationic lipid, LNP is a lipid nanoparticle) siRNA targeting TAMs, and anti-tumor response is obtained in the same tumor model. The anti-tumor therapeutic response was obtained through the silencing of STAT3 and HIF-1α, which resulted in an increase in the level of infiltrated macrophage (CD11b^+^ cells) into the tumor microenvironment as well as a tendency to increase the concentration of M1 macrophages (CD169^+^ cells). The treatment also resulted in reversing the pro-tumorous functions of TAMs -mainly angiogenesis and tumor cell activation.

### Other Non-coding RNAs Affect the Occurrence and Development of Tumor

Although the association of other ncRNAs such as piRNAs, snRNAs and snoRNAs with tumor angiogenesis has not been reported, they also play important roles in the development of tumors. Common other ncRNAs affect the occurrence and development of tumor are shown in Table 5.

**TABLE 5:** Other ncRNAs affect the occurrence and development of tumors.

**NcRNAs**	**The relationship between other ncRNAs and tumor development**	**References**
piR-1245 (piRNA)	piR-1245 acts as an oncogene and promotes the development of tumors. Its direct target is the tumor suppressor gene ATF3, BTG1, DUSP1, FAS, NFKBIA, UPP1, SESN2, TP53INP1 and MDX1.	Weng et al., 2018
U1 snRNA (snRNA)	There is a highly repetitive A > C mutation at the third base of U1 snRNA. The mutation leads to the formation of new splice junctions and changes the splicing pattern of multiple genes, leading to abnormal splicing in cancer.	Shuai et al., 2019
SNORD50A-SNORD50B (snoRNA)	In KRAS mutant tumor cells, the deletion of SNORD50A and SNORD50B promoted tumorigenesis, and the deletion of SNORD50A and SNORD50B and KRAS mutation coexisted significantly in multiple tumor types.	Siprashvili et al., 2016

Both PIWI protein and piRNA are mainly expressed in germ-line cells and abnormally expressed in a variety of cancer cells. Weng et al. (2018) found that piR-1245 is overexpressed in colorectal cancer, and the overall survival of patients with piR-1245 overexpression is significantly shortened. PiR-1245 acts as an oncogene and promotes tumor development by targeting tumor suppressor genes such as ATF3, BTG1, DUSP1, FAS, NFKBIA, UPP1, SESN2, TP53INP1, and MDX1.

U1 snRNA, as one of the most abundant ncRNAs in human cells, has a high recurring A > C somatic mutation at the third base (Shuai et al., 2019). This mutation changes the splicing pattern of multiple genes, including known cancer driver factors, accounting for the mechanism of abnormal snRNA in cancers.

Siprashvili et al. (2016) compared 5,473 pairs of tumor-normal genome pairs. They found that in 12 common cancers, 10–40% of snoRNA loci were deleted, the deletion of snoRNAs promoted tumorigenesis, but the mechanism is still unknown.

Piwi-interacting RNAs, snRNAs, and snoRNAs are abnormally expressed in a variety of cancer cells, and play important roles in the occurrence and development of cancers. However, their relationship with tumor angiogenesis has not yet been reported. Because tumor angiogenesis plays an indispensable role in the development of tumors, the relationship between these ncRNAs and tumor angiogenesis warrants further explored.

## Conclusion

Neovascularization is of indispensability for tumor development and metastasis, with multiple formation patterns and complicated regulatory mechanisms. The main physiological processing of tumor angiogenesis is as follows: One of the critical outcomes of rapid tumor growth is oxygen and nutrients absence in tumor microenvironment. Hypoxia can immediately trigger the secrete of various angiogenic factors in tumor tissue. After binding to the surface receptors of ECs, these cytokines involve in promoting the proliferation and directional migration of ECs. New sprouts are shaped from the degradation of subendothelial basal membrane afterward. Upon the stimulation of several growth factors, ECs could sharply proliferate, cross bloody sprouts and move forward to neoplasm location. Next, the newly-formed ECs are able to produce massive adhesion molecules, which specifically connect with the original ECs and bridge the stretch of vascular sprouts. Consequently, a complete vessel network has established as a result of interaction between the newly-formed ECs, vascular extracellular matrix and stromal cells. In summary, the proliferation, migration and invasion of ECs and microtubule formation induced by pro-angiogenic factors are significant for tumor angiogenesis process.

**FIGURE 3:**
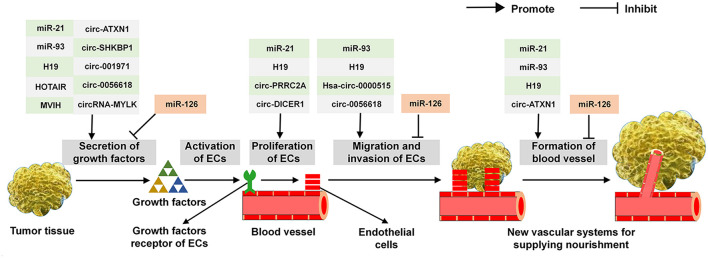
The functions of common ncRNAs on regulating the critical step in tumor angiogenesis.

As described above, a variety of ncRNAs exert multi-roles in the secretion of angiogenic cytokines, the proliferation, migration, invasion of ECs and the establishment of vascular system. Common ncRNAs that regulate the critical steps in tumor angiogenesis are shown in Figure 3. For example, it has been demonstrated that miR-21, miR-93, H19, HOTAIR, MVIH, circ-ATXN1, circ-SHKBP1, circ-001971, circ-0056618, and circRNA-MYLK are able to effectively modulate the expression of VEGF. In addition, miR-21, H19, circ-PRRC2A, and circ-DICER1 are proved to involve in the proliferation of ECs. Similarly, miR-93, H19, Hsa-circ-0000515 and circ-0056618 can affect the course of ECs migration and invasion. Moreover, regarding the establishment of vascular system, miR-21, miR-93, H19, and circ-ATXN1 play an important role in this process. For the multi-functions of ncRNAs in tumor angiogenesis, miR-21, miR-93, circ-ATXN1, and circ-0056618 participate in regulating several key aspects of this course and H19 involve in the whole processing. Of note, miR-126 exerts an adverse effect on tumor angiogenesis: it inhibits the production of VEGF, the migration and invasion of ECs and eventually abates the vascular system.

## Future Perspectives

Although the biological functions and mechanisms of ncRNA in regulating tumor angiogenesis still need to be further investigated, novel advances in past several years have been achevied in exploring the regulatory role of ncRNAs in tumor angiogenesis. For example, ncRNAs accumulation specificly in some tumor cells can be exploited to develop new medical surveillance technologies, potentially allowing faster and more accurate detection of tumor initiation and progression. In addition, RNA sponges, specific interfering molecules targeting ncRNAs that function as proto oncogenes and ncRNAs that serve as tumor suppressors have been synthesized for anti-tumor angiogenesis therapy. Therefore, the emerging relationship between ncRNAs and tumor angiogenesis opens up new horizons for its diagnosis and treatment. Notably, although researchers have investigated multiple methods to transform ncRNAs to applicable biomarkers or the targeted drugs, several problems still need to overcome in present, such as the instability of RNA itself, the indetermination of temporal and spatial expression of ncRNAs, and the unknown other side effects. In short, ncRNAs regulate tumor angiogenesis and could be targets of novel drug development for cancer treatment. One can hope that in the near future, the relationship between ncRNAs and angiogenesis will be better understood, with their value provided original and potential strategies for cancer management.

## Author Contributions

PS, DD, and WG conceived and designed this study. XS and YG created the figures and tables. XS and DD wrote the draft of the manuscript. PS and WG reviewed and edited the manuscript. All authors read and approved the final manuscript.

## Conflict of Interest

The authors declare that the research was conducted in the absence of any commercial or financial relationships that could be construed as a potential conflict of interest.

## Publisher’s Note

All claims expressed in this article are solely those of the authors and do not necessarily represent those of their affiliated organizations, or those of the publisher, the editors and the reviewers. Any product that may be evaluated in this article, or claim that may be made by its manufacturer, is not guaranteed or endorsed by the publisher.
